# Acute pancreatitis in pregnancy: a 10-year, multi-center, retrospective study in Beijing

**DOI:** 10.1186/s12884-022-04742-8

**Published:** 2022-05-17

**Authors:** Tingting Zhang, Guoxing Wang, Zheng Cao, Wenyang Huang, Hongli Xiao, Hongtao Wei, Junli Lu, Ruixia Liu, Chenghong Yin

**Affiliations:** 1 Department of Internal Medicine, Beijing Obstetrics and Gynecology Hospital, Capital Medical University, Beijing, China; 2 Department of Emergency Medicine, Beijing Friendship Hospital, Capital Medical University, Beijing, China; 3 Department of Clinical Laboratory, Beijing Obstetrics and Gynecology Hospital, Capital Medical University, Beijing, China; 4 Department of Obstetrics and Gynecology, Beijing Chao-Yang Hospital, Capital Medical University, Beijing, China; 5 Department of Gastroenterology, Beijing Friendship Hospital, Capital Medical University, Beijing, China; 6 Department of Obstetrics and Gynecology, Beijing Chao-Yang Hospital, Capital Medical University, 8 Gongren Tiyuchang Nanlu, Chaoyang District, Beijing, 100020 People’s Republic of China; 7 Department of Central Laboratory, Beijing Obstetrics and Gynecology Hospital, Capital Medical University, 251 Yaojiayuan Road, Chaoyang District, Beijing, 100026 People’s Republic of China; 8 Department of Internal Medicine, Beijing Obstetrics and Gynecology Hospital, Capital Medical University, 251 Yaojiayuan Road, Chaoyang District, Beijing, 100026 People’s Republic of China

**Keywords:** Acute pancreatitis in pregnancy, Etiology, Clinical manifestations, Maternal outcomes, Fetal outcomes

## Abstract

**Objective:**

Acute pancreatitis in pregnancy (APIP) is a rare and serious complication during pregnancy. It has acute onset and is difficult to diagnose and treat. The aim of the present study was to describe the etiology, clinical manifestations, and maternofetal outcomes of APIP.

**Methods:**

We retrospectively reviewed 32 pregnant women who were treated at three tertiary care hospitals in Beijing, China. The correlation between the causes of APIP, severity, laboratory indices, and outcomes was analyzed.

**Results:**

The most common causes of APIP were hypertriglyceridemia (56.2%,18/32) and gallstones (28.1%, 9/32). Hypertriglyceridemia-induced APIP was associated with a higher rate of severe acute pancreatitis (*P* = 0.025). Serum level of triglycerides showed a positive correlation with the severity of APIP (*P* = 0.039). The most frequent presentation of APIP was abdominal pain (93.7%, 30/32). There were no maternal or fetal deaths in our study. Apgar scores at 1 min, 5 min, and 10 min of the premature neonates was correlated with the severity of APIP of the mother (*P* = 0.022; 0.002; 0.002).

**Conclusion:**

High level of triglycerides may serve as a useful marker of the severity of APIP. The severity of APIP was associated with higher risk of neonate asphyxia. Appropriate timing of termination of pregnancy is a key imperative for APIP patients.

**Supplementary Information:**

The online version contains supplementary material available at 10.1186/s12884-022-04742-8.

## Introduction

Acute pancreatitis in pregnancy (APIP) is one of the rare and serious complications during pregnancy [1–3]. The reported incidence rate of APIP varies between 1/12000–1/1000 [1–3]. An increase in the incidence rate of APIP has been observed over the past decades [4–7]. Acute progression of APIP may lead to pancreatic necrosis, abscess, multi-organ dysfunction, and other adverse maternal and fetal outcomes. APIP has an acute onset and is typically difficult to diagnose and treat. According to a study, APIP may be more harmful to the fetus compared to the mother [7]. However, owing to its rarity, most published research on this subject is based on small case series. In addition, most retrospective studies had a relatively long reference time-period; therefore, these studies could not characterize the changes in APIP characteristics in recent years. Furthermore, the features of APIP may vary greatly in different geographical areas and ethnic groups [1, 2, 7, 8].

Of late, there have been great changes in the diagnosis and treatment of APIP. The early diagnosis, etiology and outcome of APIP have attracted the attention of many researchers [8–10] However, there is still a paucity of contemporary reports from China on the clinical features of APIP and the outcomes. As a relatively developed region in China, Beijing's economic development level and medical level are in the forefront of the country, and the penetration rate of medical knowledge is higher. The incidence, etiological composition, maternal and infant outcomes of APIP in Beijing may be different from other regions of China. We also can provide many experiences for the diagnosis and treatment of APIP patients. But, there is only one single center APIP retrospective study in Beijing, and it was six years ago.

In this study, we retrospectively reviewed 32 cases of APIP treated at the following three tertiary care centers in Beijing, China: Beijing Obstetrics and Gynecology Hospital; Beijing Chaoyang Hospital; and Beijing Friendship Hospital. All three hospitals are affiliated to the Capital Medical University. The Beijing Obstetrics and Gynecology Hospital is a top maternal and child health care hospital with 660 beds and the number of births exceeding 14,000 every year. The Beijing Chaoyang Hospital is an advanced general hospital, and has a well-known emergency medicine clinical research center. The Beijing Friendship Hospital is also an advanced general hospital, and has a national digestive system disease clinical research center. Patients with APIP generally prefer to go to obstetrics, emergency department, and gastroenterology; therefore, we selected these three hospitals for conducting this research. The aim of the present study was to describe the etiology, clinical manifestations, and maternofetal outcomes of APIP.

## Methods

### Study design

**Fig. 1:**
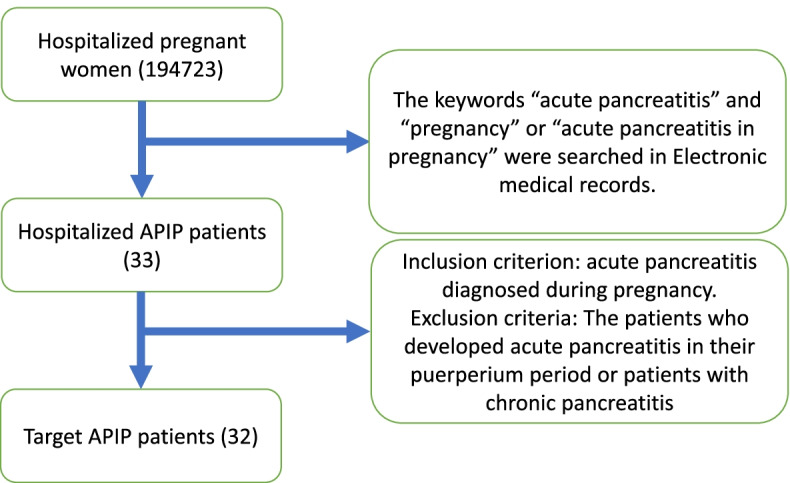
Flowchart for study population

This study was designed as a real-world, retrospective, cross-sectional, multicenter study on patients hospitalized with APIP in Beijing of China. All three hospitals agreed to participate in this retrospective study. We reviewed 194723 pregnant women who attended the Chaoyang Hospital between 2015 and 2020, and Beijing Obstetrics and Gynecology Hospital and Beijing Friendship Hospital between 2010 and 2020. The inclusion criterion was acute pancreatitis diagnosed during pregnancy. Patients who developed acute pancreatitis in their puerperium period or patients with chronic pancreatitis were excluded. The keywords “acute pancreatitis” and “pregnancy” or “acute pancreatitis in pregnancy” helped to search and collect 33 cases of APIP patients. 32 pregnant women were finally included in this study (1 Patient who developed acute pancreatitis in their puerperium period were excluded) (Fig. 1). The study was approved by the institutional review board of the Beijing Obstetrics and Gynecology Hospital. The records and data did not include potential patient identifying information, so informed consent was not required.

### Data collection

Data pertaining to the following variables were collected from the Electronic medical records (EMR): maternal age, gestational age at the time of presentation and delivery, potential causes of APIP, clinical features and complications, diagnostic tests, clinical management, and maternal and infant outcomes. We wanted to evaluate the early predictive value of routine laboratory tests for APIP severity, so we collected laboratory test data, including the biochemistry and hematologic tests data within 48 h of admission (All the data were the results of the first examination after admission).

### Definitions

The diagnosis and severity categories of APIP were according to the Atlanta Criteria and Clinical practice guideline [11, 12]. The diagnosis of acute pancreatitis requires two of the following three features: (1) Abdominal pain consistent with acute pancreatitis; (2) Serum lipase activity (or amylase activity) at least three times greater than the upper limit of normal; and (3) characteristic findings of acute pancreatitis on contrast-enhanced computed tomography (CECT) and less commonly magnetic resonance imaging (MRI) or transabdominal ultrasonography. Mild acute pancreatitis (MAP) is characterised by the absence of organ failure and the absence of local or systemic complications; Moderately severe acute pancreatitis (MSAP) is characterised by the presence of transient organ failure or local or systemic complications in the absence of persistent organ failure (< 48 h); Severe acute pancreatitis (SAP) is characterised by persistent organ failure (≥ 48 h). Organ dysfunction was defined according to the modified Marshall score, and local complications were acute peripancreatic fluid accumulation (AFPC), pancreatic pseudocyst, acute necrosis and encapsulated necrosis. APIP is also classified according to different pathogenic causes: acute gallstone pancreatitis, hypertriglyceridemic pancreatitis, and idiopathic pancreatitis [13–15]. Different pregnancy stages are defined according to the gestational age, such as the first trimester (1–12 weeks), second trimester (13–28 weeks), and the third trimester (from 29 weeks to before delivery). Preterm birth was defined as a neonate at less than 37 weeks of gestation but equal to or more than 28 completed weeks. We classified Apgar scores into three groups: low (Apgar 0–3), intermediate (Apgar 4–6), and normal (Apgar 7–10) [16]. The Apgar score was used to evaluate the degree of neonatal asphyxia, with 0 ~ 3 indicating severe asphyxia and 4 ~ 7 indicating mild asphyxia.

### Statistical analysis

All data analyses were performed using IBM SPSS 25.0. Categorical variables are presented as frequency (%) and between-group differences assessed using the Chi-squared test or the Fisher exact test, as appropriate. Normally distributed continuous variables are presented as mean ± standard deviation (SD) and between-group differences assessed using Student's t test or one-way analysis of variance. Non-normally distributed continuous variables were expressed as median and quartile (P25, P50) and analyzed using non-parametrical test. We used Reverse Kaplan–Meier method to analyze mean / median follow up time. All tests were two-tailed, and *P* values < 0.05 were considered indicative of statistical significance.

## Results

### Demographics

During the study reference period, we reviewed a total of 194723 pregnant women. According to the inclusion and exclusion criteria, 32 pregnant women were finally included in this study. The incidence rate of APIP in our study is 1.6/10000. The mean / median follow up time of MAP, MSAP and SAP patients are 8.6/8.0, 11.3/10.0 and 13.6/10.5 (Table 1). The mean maternal age was 30.6 ± 3.8 years (range 20–39). The average gravidity and parity was 2.0 ± 1.1 and 0.3 ± 0.5. The mean gestational age was 33.2 ± 5.4 weeks with majority of the episodes occurring in the third trimester (90.6%, 29/32)(Table 2). Approximately 28.1% (9/32) of the patients were multiparous, and 71.8% (23/32) were nulliparous. Two patients (6.3%) experienced recurrences during the same pregnancy. Moreover, 4 patients underwent IVF-ET (in vitro fertilization and embryo transfer) and 3 patients had twin pregnancies.

**Table 1:** The mean/median follow up time of acute pancreatitis in pregnancy

Severity of APIP n (%)	mean/median	MAP	MSAP	SAP
		*P*^a^ value	*P*^a^ value	*P*^a^ value
MAP	8.6/8.0		0.667	0.146
MSAP	11.3/10.0			0.350
SAP	13.6/10.5			
Total 32 (100)	10.5/10.0			

^a^ Log Rank (Mantel-Cox)

*MAP* mild acute pancreatitis, *MSAP* moderately severe acute pancreatitis, *SAP* severe acute pancreatitis

**Table 2:** Distribution of episodes of acute pancreatitis by trimester

Trimester n (%)	Age^a^, years (mean ± SD)	Gravidity (mean ± SD)	Parity (mean ± SD)	GA, weeks (mean ± SD)
1st 0 (0)				
2nd 3 (9.375)	33.0 ± 3.6	2.7 ± 0.6	0.7 ± 0.6	20.3 ± 6.4
3rd 29 (90.625)	30.3 ± 3.8	2.0 ± 1.1	0.2 ± 0.4	34.5 ± 3.2
Total 32 (100)	30.6 ± 3.8	2.0 ± 1.1	0.3 ± 0.5	33.2 ± 5.4

^a^ Normally distributed continuous variables

*GA* gestational age, *SD* standard deviation; Gravidity: the number of pregnancies; Parity: the number of parturitions

### Etiology

The most common causes of APIP were hypertriglyceridemia (56.2%, 18/32) and gallstones (28.1%, 9/32). The other causes of APIP were idiopathic (12.5%, 4/32) and gallstone complicated with hypertriglyceridemia (3.1%, 1/32) (Table 3). Among all the patients, 56.3% (18/32) had mild acute pancreatitis (MAP), including 7 patients with hypertriglyceridemia and 11 patients without hypertriglyceridemia. The observed between-group difference in the incidence of MAP was statistically significant (*P* = 0.025), which suggested that patients without hypertriglyceridemia tended to have milder clinical manifestations.

**Table 3:** Etiology and clinical characteristics of acute pancreatitis in pregnancy

	Other causes			HTG-AP	*P* value^a^
	Gallstone	Idiopathic	Mix		
Number n (%)	9 (28.1)	4 (12.5)	1 (3.1)	18 (56.3)	
Severity of APIP					
MAP	7	4	0	7	0.025*
MSAP + SAP	2	0	1	11	
Gestational diabetes mellitus	2	1	0	5	1.000
Fatty liver disease	2	0	0	3	1.000
Pleural effusion	2	0	0	5	0.426
Ascites	2	0	1	7	0.446
Pelvic effusion	1	0	0	3	0.613
Localized complications	1	0	0	3	0.613
Organ dysfunction	2	0	1	11	0.036*
Intrahepatic cholestasis of pregnancy	0	1	1	0	0.183
Timing of diagnosis ≥ 24 h	5	1	0	5	0.465
Length of stay ≥ 10 days	5	0	1	12	0.283
Amylase level > 172 U/L	8	1	1	14	0.703
Marshall score ≥ 2	2	0	1	11	0.036*

*HTG-AP* hypertriglyceridemic acute pancreatitis

^a^ Fisher test

### Clinical manifestations of APIP

The most frequent presentation of APIP in our cohort was abdominal pain (93.7%, 30/32). More than half of all patients had nausea and vomiting (71.8%, 23/32) while fever was less common (18.7%, 6/32). We compared the commonly used laboratory indices according to the severity of APIP [MAP, moderately severe acute pancreatitis (MSAP), and severe acute pancreatitis (SAP)] (Table 4). Only the level of triglycerides showed a positive correlation with the severity of APIP (*P* = 0.039). However, serum amylase, serum glucose, serum calcium, leukocyte count, total cholesterol, low-density lipoprotein cholesterol, and high-density lipoprotein cholesterol showed no correlation with the severity of APIP (Table 4).

**Table 4:** Severity of APIP and abnormality of serum indices

	MAP	MSAP	SAP	*P* value^a^
Number	18	4	10	
Age, years	30.9 ± 3.3	30.8 ± 1.7	30.1 ± 5.5	0.881
Amylase level > 172 U/L	12	3	9	0.458
White blood cell count	11	3	9	0.287
NE > 75%	14	2	10	0.074
Hyperglycemia ≥ 7.8 mmol/L	2	0	0	0.637
Hypocalcemia < 2 mmol/L	4	0	3	0.605
Hypertriglyceridemia ≥ 11.3 mmol/L	6	3	8	0.039*
Total cholesterol ≥ 5.65 mmol/L	14	4	8	0.838
Low-density lipoprotein cholesterol > 3.3 mmol/L	5	1	6	0.236
High-density lipoprotein cholesterol < 1 mmol/L	3	3	3	0.073
Timing of diagnosis ≥ 24 h	5	2	5	0.501
Length of stay ≥ 10 days	6	3	9	0.006*

^a^ Fisher test

### Maternal and fetal outcomes

There were no maternal and fetal deaths in our cohort. However, one patient asked to be discharged from the hospital and gave up treatment. Another patient requested induction of labor because she was afraid that the drugs used during the treatment would be harmful to her fetus. In our study, 59.3% (19/32) of the patients underwent emergency cesarean section owing to the medical condition. Five (18.5%) live births were diagnosed with neonatal respiratory distress syndrome (Table 5). Apgar scores at 1 min, 5 min, and 10 min of the premature neonates were evaluated in 14 cases (Table 6). We suggested that Apgar scores at 1 min, 5 min, and 10 min of the premature neonates was correlated with the severity of APIP of the mother (*P* = 0.022; 0.002; 0.002). However, the length and weight of the neonates showed no correlation with the severity of APIP (Table 7). Fetal malformations were observed in two cases; one was hypospadias, and the other was gastrointestinal malformation with congenital heart disease.

**Table 5:** Maternal and fetal outcomes

	MAP	MSAP	SAP
Number	18	4	10
Cesarean birth	10	2	9
Vaginal delivery	2	1	0
Continued pregnancy	6	0	1
Induction of labor^a^	0	1	0
Postpartum hemorrhage	6	0	1
neonatal respiratory distress syndrome	1	1	3
Maternal or fetal death	0	0	0

^a^ patient requested induction of labor

**Table 6:** Apgar scores of premature infants

	MAP	MSAP	SAP	*P* value^a^
Number	9	2	3	
1 min	9.0, 10.0	9.0, 9.5	5.0, 7.0	0.022*
5 min	10.0, 10.0	10.0, 10.0	5.0, 9.0	0.002*
10 min	10.0, 10.0	10.0, 10.0	5.0, 9.0	0.002*

^a^ non-parametrical test (Kruskal–Wallis H)

**Table 7:** the baseline data of neonates

	MAP	MSAP	SAP	*P* value
Number	15	3	9	
Length^a^, cm	45.8 ± 4.0	48.7 ± 1.2	45.8 ± 3.8	0.479
Weight^a^, g	2478.7 ± 719.2	2883.3 ± 261.6	2710.6 ± 761.4	0.573

^a^ one-way analysis of variance

## Discussion

### Summary of findings

The present study described 32 cases of APIP with the aim to characterize the clinical correlates of this disease in Beijing of China. The incidence rate of APIP in our study is 1.6/10000. The incidence of APIP in Beijing is lower than that in other regions [1, 8, 17]. The mean / median follow up time of MAP, MSAP and SAP patients are 8.6/8.0, 11.3/10.0 and 13.6/10.5 (Table 1). There is no difference in mean / median follow up time between groups, but as shown in the Table 1: the more serious the disease is, the longer the mean / median follow up time is. In our cohort, most of the events (90.6%, 29/32) presented in the third trimester, which suggests an increased incidence with increasing gestational age. Gallstone is the most commonly reported etiology of AP among pregnant women in Europe and America, followed by idiopathic, alcohol abuse, and hypertriglyceridemia-induced AP [1, 2, 18–20]. However, the most common cause of APIP in Chinese women in this study was hypertriglyceridemia (56.3%, 18/32). The results are in line with previous studies conducted in China [6, 8]. And, compared with the previously reported data, the proportion of APIP cases caused by hyperlipidemia was higher in our study [6–8, 21]. Many cases of APIP caused by hypertriglyceridemia were also reported in Japan and Korea [22–24]. Obviously, the etiological pattern of APIP is different between Asian and Western women, and there are several reasons can explain this. First, different dietary cultures during pregnancy. Although alcohol abuse is uncommon during pregnancy in China, most pregnant women tend to have high-fat diet due to local culture. Second, a number of studies have indicated that the incidence rate of gallstones is related to ethnicity. The prevalence of gallstones in Asian countries, including China, is significantly lower than that in western countries [18, 25–27]. Third, the plasma lipid levels are liable to increase during pregnancy due to the effects of estrogen, progesterone, and human placental lactogen [28]. Lipid levels in the first trimester are usually the same as pre-pregnancy and change significantly in the second and third trimesters [29]. There is usually a two to four-fold increase in plasma triglyceride level during pregnancy [30], which is normally well-tolerated (< 300 mg/dL or 3.3 mmol/L) and does not affect the mother or fetus [31], but in some high-risk women, triglyceride level may increase to an abnormally high level (more than 95^th^ centile for the age) or even severe level (> 1000 mg/dL or 11.3 mmol/L) [32]. Moreover, previous studies demonstrated that women from East and South Asia, including China, have higher levels of TG, TC and LDL than western populations [33]. Therefore, it is considered that the ethnicity is one of the risk factors of higher triglyceride level in East and South Asia pregnant women. Finally, we speculate that the rising trend of obesity worldwide may have an impact on the incidence of this disease [6]. Several studies have shown that patients with APIP caused by hypertriglyceridemia have an increased tendency to develop SAP and other complications [1, 7, 21, 31, 34]. We also observed a similar tendency in our study. We suggested that Apgar scores at 1 min, 5 min, and 10 min of the premature neonates was correlated with the severity of APIP of the mother (*P* = 0.022; 0.002; 0.002). Although recent studies suggest that the Apgar score alone cannot be considered to be evidence of or a consequence of asphyxia. However, based on population studies, Apgar scores of less than 5 at 5 and 10 min clearly confer an increased relative risk of cerebral palsy, and the degree of abnormality correlates with the risk of cerebral palsy [16].

### APIP population vs general pregnant population

In order to compare APIP pregnant women with general pregnant women, 200 pregnant women were collected from Beijing obstetrics and gynecology hospital from 2018 to 2020. After excluding the history of surgery, infection, adverse pregnancy and reproductive system diseases, 137 completely healthy pregnant women were included in our study (Table 8). There was no difference in age, gravidity and parity between APIP population and general pregnant population. However, the observed between-group difference in the gestational age was statistically significant (*P* = 0.000).

**Table 8:** APIP population vs general pregnant population

Population	General pregnant	APIP	*P* value^a^
Number	137	32	
Age, years (mean ± SD)	31.6 ± 4.9	30.6 ± 3.8	0.287
Gravidity (mean ± SD)	1.9 ± 1.0	2.0 ± 1.1	0.545
Parity (mean ± SD)	0.4 ± 0.5	0.3 ± 0.5	0.182
GA, weeks (mean ± SD)	38.7 ± 2.0	33.2 ± 5.4	0.000*

^a^ Student's t test

Gravidity: the number of pregnancies; Parity: the number of parturitions; *GA* gestational age, *SD* standard deviation

### Implication for practice of diagnosis

In our study, the initial diagnosis of 10 (31.2%) patients was different from the discharge diagnosis, and a delayed diagnosis of APIP was made for 12 (37.5%) patients. This means that the normal treatment (fluid resusscitation, enteral feeding) was missing or delayed in most cases, which could also influence the presence of complications and severity. Several factors can explain this phenomenon. First of all, enlargement of the uterus displaces the pancreas posteriorly, and some APIP patients may not have the typical clinical manifestation of upper abdominal pain. The clinical manifestations of nausea and vomiting are similar to various physiological or pathological manifestations during pregnancy. Moreover, inflammation in the pancreas can induce uterine contraction; thus, the abdominal discomfort is liable to be mistaken for abdominal discomfort related to labor. Second, pregnancy will affect the changes and interpretation of various blood and biochemical indices. In our study, 25% (8/32) of patients had an increase in serum amylase less than three times the normal value, and the increase in serum amylase was not related to the severity of APIP. Studies have shown that compared with serum amylase, serum lipase has a higher sensitivity and a larger diagnostic window [35]. In case of hyperlipidemic acute pancreatitis, lipase offers a better diagnostic accuracy (91.8%) than amylase (40.3%) [36]. Moreover, the research of Lichun Zhang et al. have shown that lipase, Neutrophil–lymphocyte ratio, gamma-glutamyl transpeptidase, high-density lipoprotein can serve as a panel of factors to predict APIP [37]. Regrettably, serum lipase levels were not tested in most of the patients in this study. Thus, based on previous studies, we strongly recommend that serum lipase should be added to the routine biochemical examination of patients with abdominal pain during pregnancy to facilitate the diagnosis of APIP. Third, although ultrasound is still the first abdominal imaging method for APIP patients, the findings are greatly affected by gastrointestinal gas and the posture of pregnant women. Thus, use of ultrasound alone may not be adequate for accurate assessment of APIP. Magnetic resonance imaging (MRI) and Magnetic resonance cholangiopancreatography (MRCP) without iv contrast (gadolinium) should be considered in patients with indeterminate ultrasound findings [38].

### Implication for practice of management

In the present study, we found that the clinical management of APIP has remained almost the same in the past decade. Treatment of APIP requires multidisciplinary collaboration involving specialists from gastroenterology, radiology, obstetrics, general surgery, neonatology, and even intensive care departments to develop personalized treatment plans. Owing to the lack of standardized guidelines for APIP, the treatment is generally conservative and similar to that administered to non-pregnant patients with acute pancreatitis.

In our study, 55.5% of gallstone-induced APIP patients had a history of gallstones and 27.7% of hypertriglyceridemia-induced APIP patients had hyperlipidemia during pregnancy (one of which had a history of hyperlipidemia for two years before pregnancy). Therefore, we recommend primary prevention for high-risk patients (history of gallstones, hyperlipidemia or hyperlipidemia during pregnancy, and BMI ≥ 28 kg/m^2^) before and during pregnancy. For pregnant women with a history of gallstones, we recommend abdominal ultrasound examination during the pre-pregnancy counseling. Timely treatment of patients who are found to have gallstones can help prevent APIP. Patients with hyperlipidemia should stop taking lipid-lowering drugs during pregnancy owing to the lack of definitive evidence of their safety during pregnancy [32]. However, they should be fully informed of the possible complications and treatment methods during pregnancy. It is recommended to improve their lifestyle including avoidance of excessive weight gain during pregnancy [31].

The management of APIP is complicated by the decision-making regarding the timing and route of termination of pregnancy (induction of labor or cesarean section or vaginal delivery) [39]. Pregnancy termination can also be regarded as a key to achieve cure of AP [40]. In our study, pregnancy was terminated in 50% (9/18) of MAP patients and all MSAP and SAP patients (92.8%, 13/14), except for one patient who became ill at 23 weeks gestation. Based on the treatment of 32 patients, we have summarized some recommendations for termination of pregnancy: (1) Women who agree to the use of fetotoxic medication for pancreatitis treatment or voluntarily terminate their pregnancy; (2) Stillbirth, fetal malformations, and severe fetal distress; (3) Patients who are in the third trimester and whose condition deteriorates after 24–48 h of treatment; (4) MSAP and SAP patients. If conditions permit, vaginal delivery should be preferred as it can help avoid infections associated with cesarean delivery. However, for APIP patients whose condition is still worsening after 24 to 48 h of active treatment (e.g., no improvement in paralytic intestinal obstruction), cesarean delivery should be undertaken immediately to ensure maternal and fetal safety [31].

No maternal or fetal deaths occurred in our study. This observation is in agreement with previous studies [2, 6]. The good outcomes in our cohort are likely attributable to the improvement in maternal and neonatal intensive and supportive care that have occurred during the past decade in China.

### Implication for further research

The incidence rate of hyperlipidemic acute pancreatitis in pregnancy increased gradually. According to the existing research results, we reasonably speculate that serum lipase may be more dominant in early diagnosing and predicting the severity of APIP than serum amylase. However, there are few relevant studies on this aspect, and serum lipase levels were not tested in most of the patients in our study. More studies are warranted for further elucidation the role of serum lipase in APIP。And we will also devote ourselves to this research in the future.

### Strength and limitations

Like all retrospective studies, some limitations should be noted when interpreting.

the results. First, because of de-identification of all personal information of patients, follow-up data of APIP patients after their discharge from the hospital were not available. Second, some laboratory indices (such as serum lipase level) were not assessed for all patients. Third, due to the low incidence of APIP, there were only 32 cases in our study. And the number of premature infants in our study was small too, which may lead the results less meaningful. However, the main strength of this study is that the study sample was drawn from a large dataset of pregnant women and so might add valuable practical information to the global knowledge of APIP. In addition, in this article, we share some experience in the prevention, diagnosis and treatment of APIP patients, hoping to provide some implications.

## Conclusion

This study analyzed the clinical characteristics, diagnosis and treatment of APIP patients in Beijing of China. Recent advances in diagnosis and treatment of APIP have led to a decrease in maternal and fetal mortality. Recent studies have substantially improved our understanding of acute pancreatitis in pregnancy, and we look forward to further advances.

## Supplementary Information

**Additional file 1: Data 1.** The basic information of fetus of APIP patients.

## Data Availability

The data is availability. The datasets generated and/or analysed during the current study are not publicly available due some reasons but are available from the corresponding author on reasonable request. The data that support the findings of this study are available from Beijing Obstetrics and Gynecology Hospital but restrictions apply to the availability of these data, which were used under license for the current study, and so are not publicly available. Data are however available from the authors upon reasonable request and with permission of Beijing Obstetrics and Gynecology Hospital.
